# New Exploration of Therapeutic Targets for Radiation Pneumonitis: Comparative Analysis of Molecular Pathways in Radiation-Induced and LPS-Induced Pneumonitis

**DOI:** 10.7150/ijms.124332

**Published:** 2025-10-24

**Authors:** Siyi Niu, Zhen Li, Nan Wang, Guoliang Xue, Huiwen Xue, Qian Hong, Wei Xu, Zhigang Wei, Xin Ye, Qi Xie

**Affiliations:** 1 Department of Oncology, Lung Cancer Center, The First Affiliated Hospital of Shandong First Medical University & Shandong Provincial Qianfoshan Hospital, Shandong Lung Cancer Institute, Jinan, China, 250014.; 2 School of Preventive Medicine Science, Shandong First Medical University & Shandong Academy of Medical Sciences, Jinan, China, 250117; 3 Department of Clinical Pharmacy, The First Affiliated Hospital of Shandong First Medical University & Shandong Provincial Qianfoshan Hospital, Jinan, China, 250014.; 4 Cheeloo College of Medicine, Shandong University, Jinan, China, 250033.; 5 Shandong Provincial Lab for Clinical Immunology Translational Medicine in Universities, Jinan, China, 250014.

**Keywords:** radiation pneumonitis (RP), radiation induced lung injury (RILI), lipopolysaccharide (LPS)-induced pneumonitis, therapeutic targets, molecular pathways

## Abstract

Radiation pneumonitis (RP) is a common complication of radiotherapy that significantly limits the tolerable radiation dose, thereby compromising treatment outcomes. In severe cases, it can become life-threatening. Currently, the management of RP relies primarily on glucocorticoids. However, this approach is associated with several drawbacks, including markedly increased risks of immunosuppression and infection, metabolic disturbances, musculoskeletal injury, gastrointestinal adverse effects, and most importantly- a potential compromise of antitumor efficacy. Moreover, current treatments for RP and radiation induced pulmonary fibrosis remain unsatisfactory, underscoring the need for mechanistic studies and the exploration of novel therapeutic targets and strategies. RP and lipopolysaccharide (LPS) -induced pneumonitis, originating from Gram-negative bacteria, represent two distinct forms of pulmonary inflammation. We compare their molecular mechanisms—with both shared pathways and key distinctions despite clinical similarities—and explore diverse therapies, including anti-inflammatory responses, antioxidant defenses, gut microbiota regulation, cell death modulation, mitophagy enhancement, among others. In particular, we highlight therapies and targets that have shown efficacy in LPS-induced pneumonitis but have not yet been investigated in the context of RP. These insights may offer valuable guidance for both clinical management and fundamental research on RP.

## Introduction

Radiation pneumonitis (RP) is a common side effect of radiotherapy, frequently arising after the irradiation of malignant tumors in the chest and breast, particularly in the treatment of lung cancer. Radiation exposure inflicts damage on cells in lung, resulting in DNA damage and an excessive accumulation of reactive oxygen species (ROS) 1. This cascade of events subsequently triggers an inflammatory response, ultimately causing damage to alveolar epithelial cells and impairment of adjacent endothelial tissue 2. The diagnosis of RP typically relies on the patient's treatment history and the manifestation of a spectrum of clinical symptoms 3. Common symptoms often include dyspnea (shortness of breath), a dry cough devoid of sputum, low-grade fever, chest pain, and overall feelings of malaise 4. RP predominantly manifests within the irradiated region and seldom affects distant lung tissues 1. This condition significantly constrains the permissible radiation dose, thereby impacting treatment efficacy, and in severe instances, can pose a life-threatening risk to the patient 5.

Hospital-acquired pneumonia is predominantly caused by Gram-negative bacilli, with common culprits including *Klebsiella pneumoniae*,* Escherichia coli*,* Proteus*,* Haemophilus influenzae*,* Pseudomonas aeruginosa*, etc. 6. Lipopolysaccharide (LPS), a major constituent of the Gram-negative bacterial cell wall, plays a pivotal role in mediating the pathological progression of pneumonitis and is classified as an endotoxin. The structure of LPS comprises hydrophilic polysaccharide chains referred to as O-antigens, an oligosaccharide core, and lipid A, which is highly toxic 7. Lipid A, serving as the biologically active core and primary toxic component of LPS, acts as a potent trigger for innate immune responses. LPS has the potential to trigger a spectrum of acute pulmonary conditions, such as pneumonitis and acute lung injury (ALI), and it may also contribute to the development of various chronic diseases 8, 9. ALI is marked by the aggregation of white blood cells, epithelial cell damage, pulmonary edema, heightened alveolar permeability, and extensive diffuse alveolar damage 10.

Currently, the management of RP relies primarily on glucocorticoids. However, this approach is associated with several drawbacks, including markedly increased risks of immunosuppression and infection, metabolic disturbances, musculoskeletal injury, gastrointestinal adverse effects, and most importantly- a potential compromise of antitumor efficacy. Identifying new therapeutic targets is particularly crucial. This review examines the similarities and differences in the pathogenic mechanisms between RP and LPS-induced pneumonitis, summarizing potential therapeutic strategies and shared molecular targets for both conditions. The goal is to identify new effective drugs for RP.

## Clinical management

### Clinical management for RP

There exists an incubation period for the clinical manifestations of RP, typically spanning from several weeks to months following radiation exposure. Although overt symptoms of pneumonitis may not be evident at the conclusion of radiotherapy, subtle pathological alterations can be identified within the lung tissue. Upon confirmation of a RP diagnosis, the most immediate and critical intervention is the cessation of radiotherapy. To ensure standardized treatment protocols, patients are evaluated and categorized based on the severity of their pneumonitis. The Radiation Therapy Oncology Group (RTOG) and the Common Terminology Criteria for Adverse Events (CTCAE) are two widely adopted grading systems utilized in clinical practice to assess radiation toxicity 11.

Glucocorticoids serve as the primary treatment for RP, renowned for their robust anti-inflammatory and antifibrotic effects. For patients exhibiting mild symptoms, it is advisable to contemplate initiating treatment with non-steroidal anti-inflammatory drugs (NSAIDs) or inhaled corticosteroids as a viable therapeutic option 4. For patients who exhibit intolerance to steroids, other immunosuppressive agents such as azathioprine and cyclosporine may be considered as potential alternatives. However, such applications have been documented only in case reports, and there currently exists a dearth of robust clinical trial data to substantiate their efficacy in the management of RP 12.

Theoretically, RP is considered a sterile inflammatory condition; however, patients are at heightened risk of secondary lung infections due to the immunosuppressive effects that may arise from glucocorticoids therapy. Upon the earliest indication of infection, empirical anti-infective therapy should be initiated without delay. Additionally, the antibiotic regimen should be promptly adjusted based on the results of sputum culture and drug sensitivity testing 13. Particular vigilance is warranted for potential infections with *Pneumocystis* and other pulmonary fungal pathogens 14. Furthermore, several medications have demonstrated the efficacy in inhibiting fibrosis, thereby decelerating the progression of RP 15. Moreover, the treatment plan should be meticulously tailored to accommodate the diverse clinical presentations of each patient. The prevalent symptoms of RP encompass dyspnea 16, which can range from mild to severe, and a persistent dry cough devoid of sputum. Symptomatic interventions, such as cough suppression, expectorant therapy, and oxygen administration, may be employed as needed to alleviate these symptoms.

### Clinical management for LPS-induced pneumonitis

LPS constitutes a crucial structural element of the cell wall in Gram-negative bacteria, functioning as a potent endotoxin and playing a pivotal role in the pathophysiological mechanisms underlying bacterial infections. The prevalent gram-negative bacteria responsible for triggering pneumonitis primarily encompass *Haemophilus influenzae*, *Klebsiella pneumoniae*, *Escherichia coli*, *Pseudomonas aeruginosa*, and *Acinetobacter baumannii*, among others 6. The current primary treatment strategies are centered around anti-inflammatory and anti-infective interventions. Among these, anti-infective therapy is the core, necessitating the selection of appropriate antibiotics based on the specific pathogen and its antibiotic sensitivity characteristics 17, 18. Glucocorticoids are the preferred anti-inflammatory agents, owing to their demonstrated efficacy in mitigating lung inflammation and fibrosis 19. Beyond these primary treatments, supportive measures like oxygen therapy will be provided. Should it be required, immunomodulators can be administered to adjust the immune system, effectively diminishing the exaggerated inflammatory response. Moreover, antioxidants may be employed to mitigate oxidative stress and preserve the structural integrity of lung tissue 20. In addition, embracing prophylactic vaccination serves as a foresighted preventive strategy 21-23. Endotoxin is a key instigator of cytokine storms in sepsis. Recent studies have elucidated that polymyxin B binds to endotoxin and effectively inactivates it 24. For those battling severe acute respiratory distress syndrome (ARDS) alongside multiple organ dysfunction syndrome (MODS), hemoperfusion with polymyxin B-embedded fibers could potentially yield considerable therapeutic benefits 25. Nevertheless, the efficacy of this therapeutic approach necessitates further validation through a substantial number of trials to definitively elucidate its clinical utility.

### Similarities and distinctions

Both RP and LPS-induced pneumonitis typically necessitate anti-inflammatory treatment with glucocorticoids. LPS-induced pneumonitis generally requires anti-infective therapy, whereas RP may also warrant antibiotic treatment if complicated by an infection. Additionally, antioxidant therapy can be employed as needed to mitigate oxidative stress 26. RP may further benefit from antifibrotic therapies designed to counteract and diminish the fibrotic processes impacting lung tissue. In the management of RP, anti-fibrotic agents represent a pivotal therapeutic strategy for counteracting progressive pulmonary damage **(Table 1)**. These pharmacologic interventions specifically target radiation-induced fibrogenesis. By inhibiting molecular pathways driving fibroblast activation and collagen dysregulation, anti-fibrotic therapies such as pirfenidone and nintedanib demonstrate clinical potential to attenuate radiation-triggered tissue stiffening while preserving alveolar-capillary gas exchange capacity 27, 28. Current clinical guidelines increasingly highlight the integration of antifibrotic drugs into multimodal therapies, acknowledging their role in preventing the insidious progression from inflammatory pneumonitis to irreversible pulmonary fibrosis. In conclusion, while LPS-induced pneumonitis treatment primarily emphasizes bacterial eradication via antimicrobial therapy, RP management focuses on inflammatory suppression, symptom alleviation, and prevention of progressive lung damage.

The clinical management of RP also presents emerging challenges. The use of glucocorticoids, while beneficial, may precipitate a spectrum of side effects, including hyperglycemia, hypertension, osteoporosis, and weight gain 19. Notably, glucocorticoids may compromise immune system homeostasis, consequently attenuating immunotherapy efficacy in tumor treatment. RP, when faced with the daunting challenge of extensive pulmonary fibrosis, encounters a paucity of effective treatments 29. Although glucocorticoid interventions are employed, they fail to reverse fibrotic changes and merely decelerate disease progression. In conclusion, current treatments for RP and pulmonary fibrosis present limitations, necessitating further mechanistic elucidation and exploration of novel therapeutic targets and strategies.

## The differences in cellular and molecular mechanism between RP and LPS-induced pneumonitis

### Cellular and molecular mechanisms for RP

**Figure 1:**
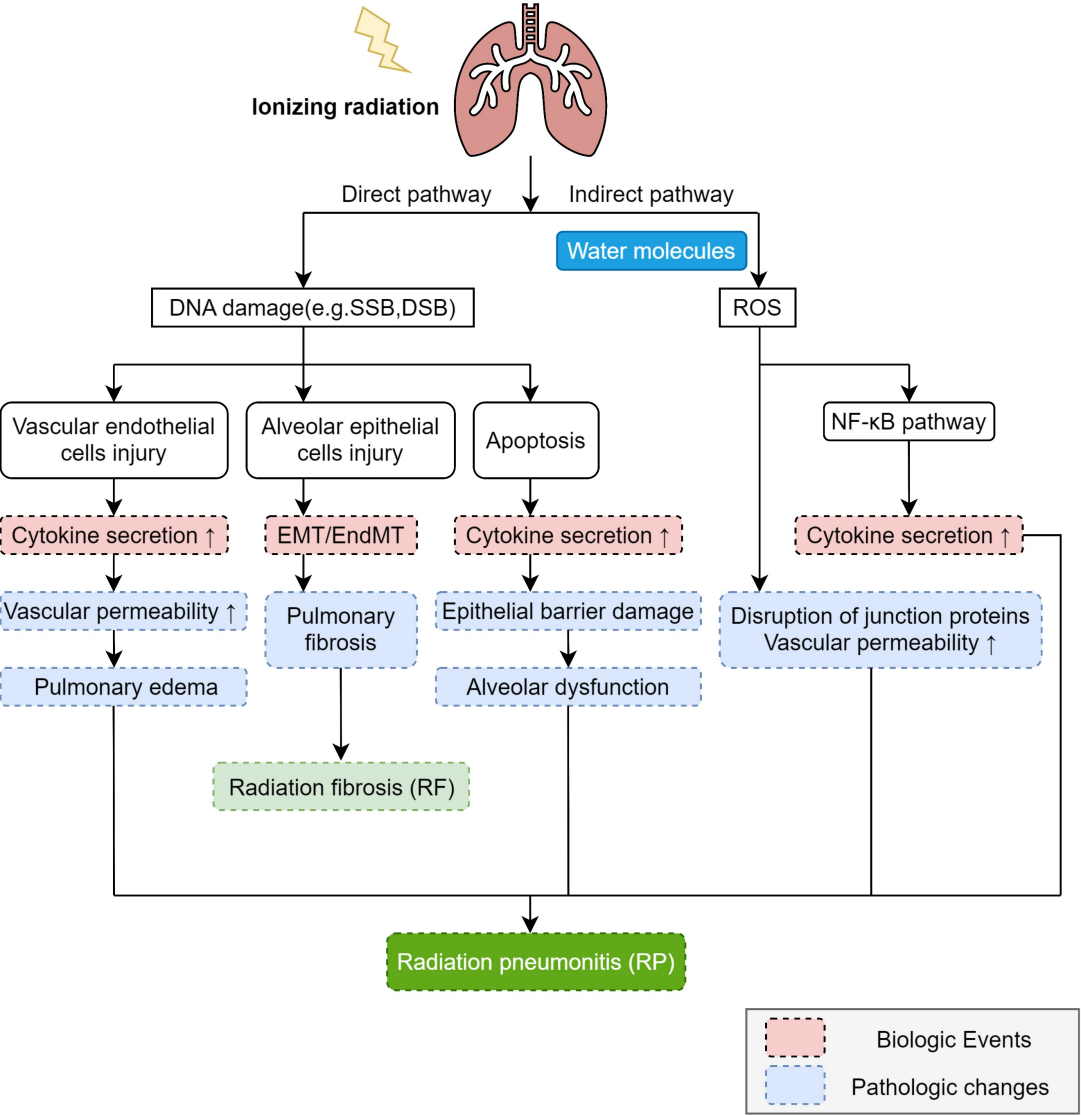
** The mechanism of RP.** Ionizing radiation induces DNA damage through direct and indirect pathways, with the direct pathway causing DNA damage (e.g., SSB and DSB) that leads to vascular endothelial cell injury and alveolar epithelial cell injury, apoptosis, increased cytokine secretion, and EMT/ EndMT, ultimately resulting in increased vascular permeability, pulmonary edema, pulmonary fibrosis, epithelial barrier damage, and alveolar dysfunction, while the indirect pathway involves ionization of water molecules in irradiated cells to generate reactive oxygen species (ROS) that damage DNA, activate the NF-κB pathway to promote cytokine secretion and participate in the development of RP, and disrupt junction proteins to further increase vascular permeability, with these radiation-induced changes collectively driving the pathogenesis of RP and RIPF.

In the pathogenesis of RP, ionizing radiation induces lung tissue damage via two primary mechanisms: direct DNA injury and the indirect production of ROS 30 **(Figure 1)**. ROS can exacerbate damage to the DNA, proteins, and lipid membranes of target cells. Subsequently, the activation of two distinct mechanisms triggers intracellular signaling, leading to the secretion of a diverse array of molecules and cytokines. This cascade ultimately promotes both inflammatory and immune responses. Ionizing radiation can directly impair deoxyribonucleic acid (DNA), such as by inducing base deletions, DNA single-strand breaks (SSBs), and DNA double-strand breaks (DSBs) 31. According to the current study, the main target cells of radiation-induced lung injury (RILI) are vascular endothelial cells and alveolar epithelial cells. The primary constituent of alveolar epithelial cells, Type I alveolar epithelial cells, do not possess the capacity for proliferation and hence exhibit relative radio-resistance 12. Nevertheless, they can still undergo necrosis or apoptosis upon exposure to certain doses of radiation 32. Radiation impairs both type II alveolar epithelial cells and vascular endothelial cells. Type II alveolar epithelial cells serve as crucial precursor cells to type I alveolar cells, and upon radiation exposure, they can initiate the excessive proliferation of fibroblasts 12. Furthermore, abnormal proliferation diminishes the secretion of alveolar surfactant substances, resulting in a reduction of alveolar surface tension. This decrement subsequently induces lung tissue edema and pulmonary atelectasis. Conversely, vascular endothelial cells exhibit heightened vascular permeability and inflammatory exudation 33.

Epithelial-mesenchymal transition (EMT) is pivotal in the pathogenesis of RILI. This process entails the gradual loss of epithelial traits and the acquisition of mesenchymal fibroblast-like characteristics by epithelial cells. Endothelial cells, as specialized epithelioid cells, undergo endothelial-mesenchymal transition (EndMT), triggering collagen deposition with subsequent declines in lung compliance and diffusion capacity. Radiation induces EMT and/or EndMT, culminating in pulmonary fibrosis 33. These cells may undergo post-radiation mitosis 34. At this stage, if the damaged DNA is not fully repaired or if the DNA damage is excessively severe, it may result in apoptosis. This ultimately compromises the alveolar barrier function and initiates an inflammatory response. Damaged cells release inflammatory cytokines that recruit inflammatory cells into the alveoli and interstitium, thereby precipitating pneumonitis 11. The severity of acute-phase RP appears dose-dependent, with higher doses correlating with more severe lung injury 35.

Ionizing radiation generates ROS through water ionization, creating an indirect pathway for DNA damage **(Figure 1)**. ROS damage not only nuclear DNA but also mitochondrial DNA (mtDNA) 1. Research indicates that ROS can activate the nuclear factor κB (NF-κB) signaling pathway- a key transcription factor regulating immune and inflammatory responses. NF-κB activation and nuclear translocation stimulate inflammation, triggering chemokine and cytokine production. ROS also disrupt intercellular junction proteins (e.g., vascular endothelial cadherin, VE-cadherin) in vascular endothelial cells, impairing the endothelial barrier. This increased vascular permeability promotes massive leukocyte migration across the endothelium and protein leakage into the alveolar lumen, ultimately causing pulmonary oedema.

Damage-associated Molecular Patterns (DAMPs) are a class of molecules that are released into the extracellular environment during cell damage or death 36. Under homeostatic conditions, DAMPs reside intracellularly. Cellular disruption from physical, chemical, or biological trauma triggers their release, enabling recognition by immune pattern recognition receptors (PRRs). In the immediate aftermath of radiotherapy, DAMPs mediate the chemotaxis of neutrophils and macrophages from circulation to sites of lung tissue damage 11. These activated cells subsequently secrete pro-inflammatory mediators including IL-6, TNF-α, and TGF-β. These cytokines exhibit dual functionality: while inducing inflammatory responses that may lead to pulmonary tissue injury, they simultaneously facilitate tissue remodeling processes 37.

### Cellular and molecular mechanism for LPS-induced pneumonitis

The LPS molecule comprises an O-specific side chain, a core region, and a lipid A component, predominantly located in the outer membrane of Gram-negative bacteria 38. LPS-induced pneumonitis mainly acts on lung epithelial cells, neutrophils, macrophages and endothelial cells through Toll-like receptor signaling pathway, cytokines and chemokines.

**Figure 2:**
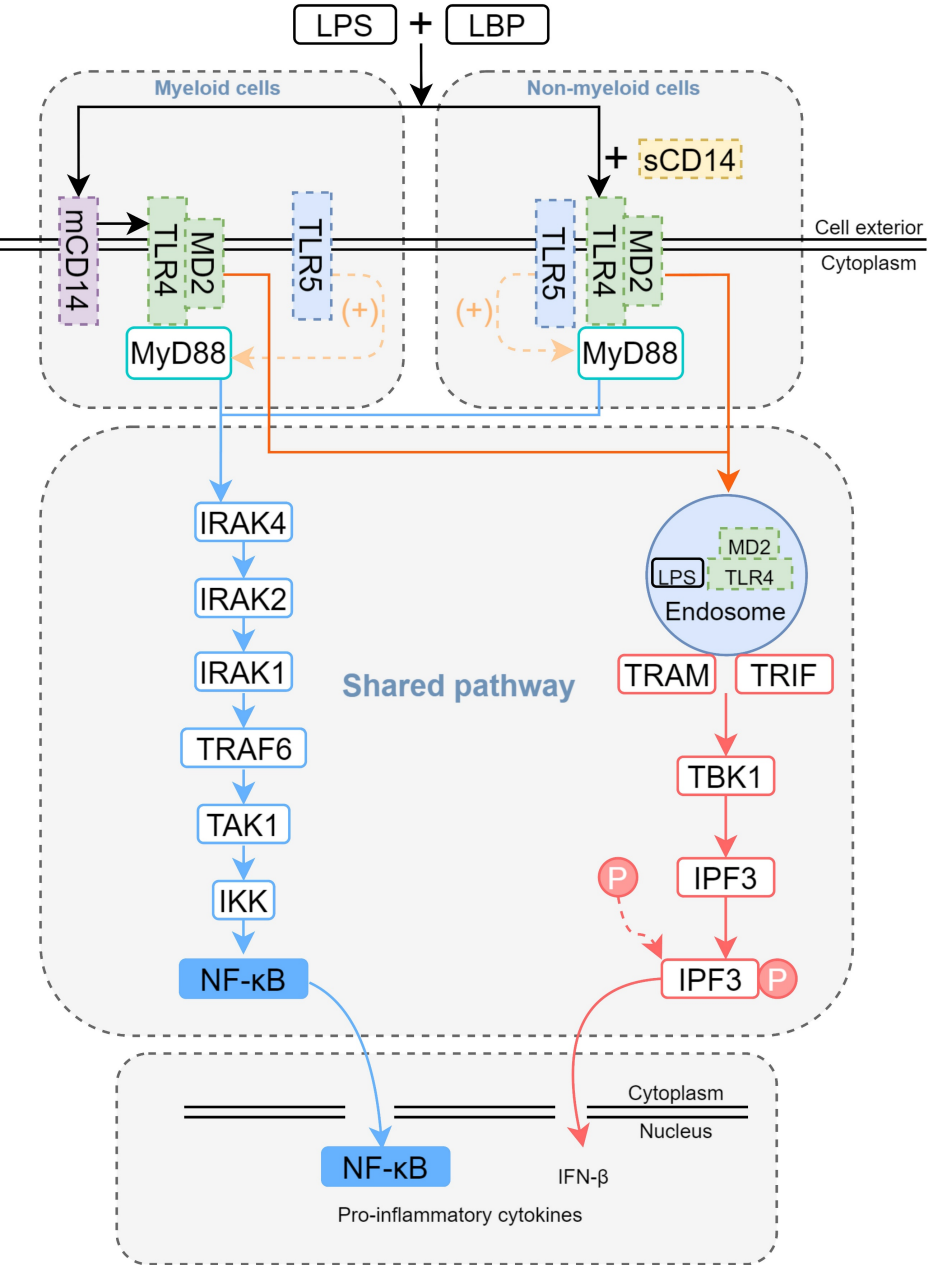
** The mechanism of LPS-induced pneumonitis.** In myeloid cells, membrane-bound CD14 (mCD14) is expressed, whereas in non-myeloid cells, CD14 exists as the soluble plasma form (sCD14). LPS forms a complex with lipopolysaccharide-binding protein (LBP) and interacts with CD14, which facilitates the delivery of LPS to TLR4. The LPS-TLR4-MD2 complex activates MyD88-dependent signaling, driving the production of pro-inflammatory cytokines via the NF-κB pathway. Simultaneously, it engages the TRIF-dependent pathway to internalize the complex, thereby inducing IFN-β generation. During LPS-induced lung injury, TLR5 may also participate. TLR5 is expressed in certain non-myeloid cells (e.g., epithelial and endothelial cells) and myeloid cells (e.g., alveolar macrophages and neutrophils). The presence of TLR5 promotes a bias toward the MyD88-dependent pathway in TLR4 signaling.

LPS engages with the LPS binding protein (LBP) situated external to the cell membrane, thereby forming a complex known as LPS-LBP 39. CD14 is a protein present on the cell membrane that recognizes and binds LPS 40. CD14 does not bind to Toll-like receptor (TLR) 4 directly, but rather delivers LPS to the complex formed by TLR4 and MD2. MD2 is an accessory protein that binds LPS and helps TLR4 recognize LPS 41. When LPS binds to MD2, it will lead to a conformational change of MD2, which in turn activates the intracellular signaling domain of TLR4 and initiates a series of signal transduction **(Figure 2)**. In the TLR4/MD2 complex, MD2 serves as a bridge, connecting LPS to the host's immune response. Without MD2, TLR4 is unable to effectively recognize LPS, making MD2 an important component of the innate immune system's defense against Gram-negative bacterial infections 42. CD11b is an integrin that plays an auxiliary role in the binding of LPS to TLR4. It is able to interact with the TLR4-MD2 complex and enhance TLR4 sensitivity to LPS.

Upon binding to LPS, TLR4 activates both the myeloid differentiation primary response 88 (MyD88)-and toll/interleukin-1 receptor (TIR)-domain-containing adapter inducing interferon-β(TRIF)-dependent signaling cascades 43. MyD88-dependent signaling rapidly initiates downstream activation of the mitogen-activated protein kinase (MAPK) family and NF-κB, driving the upregulation of pro-inflammatory cytokines such as TNF-α, IL-1β, and IL-6 **(Figure 2)**. Recent studies demonstrate a key role for TLR5 in LPS-mediated lung injury. Specifically, mechanistic findings reveal that TLR5 promotes assembly of the MyD88 complex, which amplifies the TLR4/MyD88-dependent signaling cascade 44 **(Figure 2)**. The functional interaction was evidenced by significantly reduced production of pro-inflammatory cytokines (30-50% decrease) in TLR5-deficient mouse models. Notably, TLR5 imparts signaling bias within the TLR4 complex, preferentially engaging MyD88-dependent over TRIF-mediated pathways for downstream signal transduction. Subsequently, the TLR4/MD-2/LPS complex is internalized via endocytosis, thereby activating the TRIF-dependent signaling pathway. Within intracellular compartments, TLR4 recruits TRIF through the adaptor protein TRAM. TRIF subsequently interacts with TRAF3 while simultaneously recruiting TBK1 and IKKε, initiating the phosphorylation of IRF3. Phosphorylated IRF3 then translocates to the nucleus, thereby promoting the transcription of IFN-β. Additionally, TRIF engages TRAF6 to activate TAK1, thereby triggering a delayed NF-κB-mediated inflammatory response **(Figure 2)**.

### Distinctions and similarities on cellular and molecular mechanism

#### Inflammatory response

Both LPS-induced pneumonitis and RP activate key inflammatory signaling pathways - notably NF-κB and p38 MAPK - which drive the recruitment and activation of inflammatory cells (primarily neutrophils and macrophages) in pulmonary tissue. These cells coordinate inflammatory responses by phagocytosing pathogens, secreting mediators, and generating ROS. Upon activation, these inflammatory cells secrete key mediators - including TNF-α, IL-1β, and IL-6 -which both amplify inflammation and exert chemotactic effects, recruiting more immune cells to pulmonary sites. This mediator release elevates vascular permeability, facilitating protein extravasation and cellular infiltration into lung tissue, ultimately triggering pulmonary edema. Mounting evidence highlights the pivotal role of NLRP3 inflammasome activation in the pathogenesis of both LPS-induced pneumonitis and RP. As a canonical PRR, NLRP3 constitutes the structural and functional core of the inflammasome complex 45. It is responsible for sensing intracellular and extracellular danger signals and initiates immune defense by activating inflammatory responses, which plays a dual role in defense against infection and disease development. NLRP3 inflammasome activation requires both a priming signal and an activating signal. In both pneumonitis models, NF-κB upregulation provides the priming signal, while ROS serve as the activating signal. These dual stimuli trigger assembly of the NLRP3-ASC-caspase-1 complex, leading to caspase-1 activation. Active caspase-1 processes and releases IL-1β/IL-18, which recruit neutrophils -culminating in alveolar damage and edema. NLRP3 activation dynamics differ between the two pneumonitis models. LPS rapidly induces NLRP3 expression via NF-κB activation through the TLR4-MyD88/TRIF pathway 46, 47. In contrast, RP indirectly activates NF-κB through ROS 48. Furthermore, ionizing radiation triggers mitochondrial damage, releasing mtROS and mtDNA that directly activate NLRP3 **(Table 2)**.

#### Oxidative stress

ROS production plays a critical role in innate immune defense against invading pathogens, primarily through its potent antimicrobial activity 49. However, oxygen free radicals induce the peroxidation of membrane lipids in cellular and subcellular organelles, and the excessive generation of ROS can lead to severe damage to cellular structure and function. Both LPS-induced pneumonitis and RP can harm lung tissues through oxidative stress. LPS activates the NF-κB signaling pathway through direct binding to TLR4 20, 49. Activation of NF-κB induces the expression of NADPH oxidase (NOX), which catalyzes the conversion of oxygen into superoxide anion (O₂⁻), leading to the generation of hydrogen peroxide (H₂O₂) and hydroxyl radicals (·OH). This process initiates oxidative stress through the production of ROS. In RP, ionizing radiation directly hydrolyzes intracellular water molecules, resulting in the substantial production of ROS 50. On one hand, ROS can activate the NF-κB signaling pathway, thereby perpetuating the generation of additional ROS. On the other hand, ROS can disrupt the mitochondrial electron transport chain, resulting in the release of even more ROS, thereby establishing a deleterious feedback loop. Persistent ROS stimulation prompts the release of TGF-β, which activates fibroblasts, fostering collagen deposition and ultimately leading to pulmonary fibrosis. The accumulation of lipid peroxides triggers iron-dependent cell death, further accelerating the fibrotic process 51. Nuclear factor erythroid 2-related factor 2 (Nrf2) is a pivotal transcription factor that plays a crucial role in the regulation of redox homeostasis. The upregulation of Nrf2 can mitigate the pro-inflammatory response orchestrated by the NF-κB transcription factor 52. Meanwhile, Nrf2 negatively regulates the activation of the NLRP3 inflammasome during the regulation of ROS. Considering the strong correlation among Nrf2, NF-κB, and NLRP3, the Nrf2/NF-κB/NLRP3 axis is regarded as an effective therapeutic approach to alleviate severe pulmonary inflammation 53 **(Table 2)**. The Nrf2/ARE signaling pathway also makes an important contribution to maintaining cellular homeostasis under oxidative stress 54, 55.

#### Death of cells

Pyroptosis, a programmed cell death mechanism, plays a critical role in the pathogenesis of both RP and LPS-induced pneumonitis. As radiation exposure and LPS stimulation activate inflammasomes (including NLRP3 and AIM2)—key molecular complexes that trigger pyroptosis—this process directly contributes to disease development in both pneumonitis types 56-60. Additionally, both LPS and radiation activate death receptors (e.g., Fas/FasL) on cell membranes, initiating caspase-8-mediated cleavage of downstream caspase-3 to execute apoptosis 61-64. Critically, ROS are also fundamentally implicated in both pathways. ROS directly activate pro-apoptotic proteins Bax/Bak, inducing mitochondrial damage and subsequent apoptotic cell death 65, 66.

The pathogenesis of both RP and LPS-induced ALI is mechanistically linked to ferroptosis** (Table 2)**. Gu et al. demonstrated that LPS stimulation activates ferroptosis, as evidenced by elevated levels of multiple ferroptosis markers 24 hours post-exposure 67. Radiation induces ferroptosis through a triad of mechanisms: accelerated iron metabolism, increased generation of ROS, and functional suppression of the system Xc⁻/glutathione peroxidase 4 (GPX4) axis 68, 69. It upregulates acyl-CoA synthetase long-chain family member 4 (ACSL4), which catalyzes the conversion of polyunsaturated fatty acids (PUFAs) to PUFA-CoA esters 70. This PUFA-CoA undergo enzymatic oxidation by arachidonate lipoxygenases (ALOXs) to generate lipid hydroperoxides (LOOHs) 71, 72. Simultaneously, radiation suppresses the function of the Xc⁻ system, reduces glutathione (GSH) synthesis, and impairs GPX4's ability to detoxify lipid peroxides 69. Accumulated lipid peroxides react with intracellular free iron (Fe²⁺) via the Fenton reaction, generating substantial ROS 70. This ultimately leads to rupture of the plasma membrane and organelle membranes, triggering cell death 73.

Although RP and LPS-induced pneumonitis exhibit substantial pathophysiological similarities, distinct regulatory mechanisms govern their cell death modalities. In RP, the principal modes of programmed cell death comprise pyroptosis, apoptosis, ferroptosis, and cellular senescence 55** (Table 2)**. These four modes interact to form a vicious cycle through oxidative stress, inflammation, and fibrosis signaling. Unlike LPS-induced pneumonitis, RP involves direct DNA damage caused by ionizing radiation, resulting in DSBs. These DSBs are then initiated and amplified through the coordinated activation of multiple DNA damage response (DDR) pathways, mediated by DDR kinases such as ATM, DNA-PK, and CHK2, along with their downstream effector molecules (e.g., p53 and NF-κB) 74, 75. Furthermore, recent studies have revealed that DNA damage triggers the overproduction of poly ADP-ribose (PAR) by PARP1 76. Consequently, DNA damage signals are amplified, initiating the apoptotic signaling cascade. Following radiation exposure, unrepaired double-strand breaks (DSBs) activate the p53/p21 pathway 77, 78, resulting in cell cycle arrest. As a result, senescence-associated secretory phenotype (SASP) factors activate neighboring fibroblasts, thereby promoting fibrosis 79. Research demonstrates that radiation dose variations lead to distinct cell death modalities: low-dose exposure predominantly induces apoptosis, while high-dose irradiation favors autophagy 80. Autophagy functions as a double-edged sword, with its outcomes dependent on the magnitude and duration of its activation. Under specific conditions, autophagy suppression can induce pulmonary inflammation 81.

LPS-induced lung injury exhibits a pathogenic nexus with necroptosis - an inflammation-dependent regulated cell death pathway. Unlike apoptosis, necroptosis serves dual roles as an executor of cellular demise and an amplifier of inflammatory signaling. This necrosis-associated process triggers substantial release of DAMPs, ultimately driving robust inflammatory cascades. Following LPS stimulation, RIPK1 and RIPK3 assemble into a kinase complex that mediates phosphorylation-dependent activation of MLKL 82. Phosphorylated MLKL, a key facilitator of necroptosis, triggers cell membrane disruption, resulting in cell demise. Recent studies have revealed that the TBK1/ IKKε signaling pathway exerts a suppressive effect on the inflammatory process. The LPS-TLR4-TBK1 axis functions as a negative regulator of necroptosis in polymorphonuclear neutrophils (PMNs), suggesting its potential as a therapeutic target for controlling PMN mortality and inflammation 83. NETosis constitutes a specialized cell-death program exclusive to neutrophils, releasing neutrophil extracellular traps (NETs) to capture and eliminate invading microorganisms 84 **(Table 2)**. Notwithstanding this antimicrobial defense, exaggerated NET may instigate tissue injury and propagate inflammatory pathology 85. In LPS-induced ALI, activated platelets drive NET formation, thereby amplifying tissue damage and inflammatory cascades 84. Significantly, DNase I-dependent NET degradation promoted removal of NET-bound proteins and conferred protection against ALI in murine models 86. Although RP is typically regarded as a sterile inflammation, evidence indicates that low-dose ionizing radiation elicits NET formation. This radiation ionizes oxygen molecules to produce ROS, which then activate neutrophil NADPH oxidase. Subsequent amplification of ROS triggers degranulation and IL-8 release. Through autocrine signaling via CXCR1/2 receptors, IL-8 further potentiates NETosis, creating a self-reinforcing loop that drives NET production 87. Signaling through DAMPs and ROS potently drives NETosis, a process that fuels chronic inflammation and fibrotic progression 86, 88.

#### Signaling pathways

While overlap exists between RP and LPS-induced pneumonitis in signaling pathways, their principal inflammatory cascades are not entirely identical** (Table 2)**. Radiation directly inflicts DNA lesions, initiating the DDR pathway as the primary early reaction. This cascade activates the ATM-Chk2-p53 axis to execute apoptotic cell death while concurrently imposing cell cycle arrest 58. Such arrest provides a temporal window for damage repair. When DNA damage proves irreparable, p53 can additionally drive cellular senescence and apoptosis. The TGF-β/Smad signaling pathway serves as a core regulator in RILI pathogenesis 59. Specifically, TGF-β1 transcriptionally activates SERPINE1 (PAI-1)-a potent pro-fibrotic mediator in stromal cells 60. Cells compromised by ionizing radiation secrete TGF-β1. This cytokine induces phosphorylation of downstream SMAD2/3 and, through p53-dependent mechanisms, upregulates PAI-1- culminating in fibrotic tissue remodeling 60. Separately, alveolar macrophages from thoracic radiotherapy patients constitutively secrete PDGF-BB 61. Concurrently, irradiated lung fibroblasts exhibit heightened chemotactic potency toward PDGF-BB, correlating with PDGFRB overexpression and potentially driving RP and radiation induced pulmonary fibrosis (RIPF) pathogenesis.

During LPS-induced pneumonitis, LPS primarily engages TLR4 receptors, initiating the TLR4/MyD88/NF-κB cascade. This signaling prompts the release of pro-inflammatory mediators (TNF-α, IL-1β, IL-6) and the production of ROS. NLRP3 inflammasome activation by ROS culminates in pyroptosis accompanied by IL-1β maturation. Evidence further reveals involvement of the USP7/MAPK14 axis in LPS-driven pulmonary injury. Functionally, USP7 catalytically stabilizes MAPK14 via its deubiquitinase activity and potentiates it signaling capacity, thereby amplifying inflammatory tissue destruction 62. Preclinical models demonstrate that USP7 inhibition alleviates LPS-induced pulmonary damage in mice. Separately, LPS-triggered endothelial barrier impairment correlates with RhoA/ROCK1/2 pathway activation. RhoA drives cellular proliferation, motility, and invasiveness through ROCK1/2-mediated protein modulation - a key mechanism underlying contraction-mediated endothelial hyperpermeability 63. LPS exposure significantly elevates RhoA and ROCK1/2 expression levels.

## Potential treatment targets for RP

RP and LPS-induced pneumonitis exhibit overlapping mechanisms while maintaining their own specific targets. Exploiting these shared mechanisms, it is possible to identify potential therapeutic agents for both conditions.

The NF-κB pathway and NLRP3 inflammasome contribute to both diseases. Amentoflavone (AF), previously shown to exert anti-inflammatory and antioxidant effects, was recently reported by Sun et al. to alleviate LPS-induced ALI by inhibiting the NLRP3/ASC/Caspase-1 axis and reducing pyroptosis 89. While the role of AF in RILI has not yet been investigated, its efficacy in mitigating LPS-induced ALI suggests potential therapeutic value for RP.

Targeting the modulation of cell death pathways, including apoptosis, pyroptosis, and ferroptosis, emerges as a promising therapeutic approach for addressing RP. Dapagliflozin (DPG) ameliorates LPS-induced lung injury by upregulating SIRT-1-mediated deacetylation to activate PGC-1α activity, suppressing the NF-κB inflammatory signaling pathway, enhancing mitochondrial antioxidant capacity, restoring the Bcl-2/Bax balance, and reducing caspase-3-dependent apoptosis 90. Currently, there is limited research exploring whether the SIRT-1/PGC-1α pathway can ameliorate radiation-induced damage. As a selective DRP1 inhibitor, the cell-permeable quinazolinone compound Mdivi-1 attenuates ALI by suppressing mitochondrial ROS (mtROS)/NLRP3 signaling, which consequently blocks M1 alveolar macrophage polarization and pyroptosis 91. These mechanistic insights support DRP1-dependent mitochondrial fission as a promising therapeutic target for RP requiring further validation. Obacunone (OB), a natural limonoid compound with demonstrated anti-inflammatory and antioxidant activities, confers protection against LPS-induced ALI by enhancing pulmonary antioxidant capacity, suppressing ferroptosis, and stabilizing Nrf2 through inhibition of ubiquitin-proteasomal degradation 92. Given the critical involvement of Nrf2 dysfunction and oxidative stress in RILI, OB's mechanisms position it as a promising therapeutic candidate for RP.

Regulating gut flora homeostasis might improve RP. A recent study revealed that alterations in gut microbiota can impact intestinal digestion and immune function. It was found that the lung microbiomes of mouse sepsis and human acute respiratory distress syndrome patients were enriched with bacteria of intestinal origin 93. Meanwhile, septic ALI can be accompanied by changes in the composition and function of gut microbiota, which might in turn affect distal organs through the gut-lung axis. Wang et al. discovered that Cordyceps militaris solid medium extract (CMME) decreased the levels of inflammatory factors and oxidative stress, reduced macrophage activation and neutrophil recruitment, and ultimately exerted a modulating effect on LPS-induced lung inflammation 94. This might be accomplished by regulating the gut flora and correcting metabolic disturbances. Radiation can modify the composition of the intestinal flora, resulting in a reduction in intestinal flora diversity 95. It has been shown that faecal microbiota transplantation (FMT) can maintain intestinal flora homeostasis and significantly enhance the survival rate of radiation-exposed mice, and this alteration might be achieved through the regulation of immune function 96. Whether this treatment by regulating gut flora homeostasis can improve RP requires experimental confirmation.

New methods for removing ROS may play a significant role in the treatment of RP. Manganese superoxide dismutase (MnSOD), a crucial member of the superoxide dismutase (SOD) family, functions as a ROS scavenger. Recent studies have demonstrated that plasmid-mediated MnSOD gene delivery (pMnSOD) presents a promising therapeutic strategy for radiation-induced dermal injury 97. Furthermore, systemic administration of MnSOD-engineered mesenchymal stem cells (MnSOD-MSCs) has shown significant efficacy in mitigating pulmonary inflammation 98. However, aerosolized delivery remains a technical challenge for such genetic therapies. Although preliminary evidence suggests the biosafety of nebulized MSC-derived exosomes and their potential to enhance pulmonary lesion resolution, the absence of double-blind clinical trials renders current therapeutic efficacy assessments inconclusive. The clinical translation of aerosolized MnSOD-MSCs inhalation delivery for pulmonary antioxidant therapy remains a persistent challenge 99.

Nanoparticles may also become effective drugs for the treatment of RP as ALI treatment. The design of nanoparticles for different applications can achieve objectives such as targeted drug delivery, improved bioavailability, extended sustained release, and minimized drug toxicity. Zhang et al. proposed a non-invasive inhalation of NPs for the safe and effective treatment of ALI. This formulation demonstrates excellent biocompatibility and exhibits a sustained-release pharmacological profile. It not only inhibits the release of cytokines but also blocks the triggering pathway of ALI, thereby reducing lung injury 100. Sun et al. discovered that curcumin-loaded ROS-responsive hollow mesoporous silica nanoparticles (Cur@HMSN-BSA) achieve specific drug release in high-ROS pneumonitis microenvironments 101. These nanoparticles effectively scavenge excessive intracellular ROS. Further studies confirmed its capability to promote macrophage polarization from the pro-inflammatory M1 phenotype to the anti-inflammatory M2 phenotype. Such ROS-responsive nanoparticles effectively avoided non-specific release of the drug and improved targeting. A novel X-ray-responsive nanocomposite, Au@mSiO2@Mn(CO)5Br (ASMB), was designed to mitigate RILI through localized release of CO and Mn²⁺ upon irradiation. CO suppresses NLRP3 inflammasome activation, reduces pyroptosis, scavenges ROS, and enhances DNA repair, while Mn²⁺ alleviates hypoxia via H₂O₂-to-O₂ conversion and downregulates fibrotic markers (e.g., TGF-β1, α-SMA). In RILI models, ASMB NPs reduced pulmonary edema, inflammation, and fibrosis while enhancing treatment outcomes, demonstrating dual therapeutic-radiosensitizing potential 102. An analysis of the transcriptomic profile of RILI in non-human primates revealed that the expression of SERPINA3, ATP12A, GJB2, CLDN10, TOX3, and LPA was significantly up-regulated, suggesting their potential as biomarkers and therapeutic targets for RILI 103.

Excessive production of NETs has been shown to promote ROS generation, which contributes to tissue damage. Limiting NETs overproduction therefore emerges as a promising therapeutic approach. *In vitro* experiments have demonstrated that knocking out CXCL2 significantly reduces NET formation and ROS production. Epigallocatechin-3-gallate (EGCG), a natural compound derived from green tea, exhibits anti-cancer, anti-inflammatory, and antioxidant properties. Experimental data reveal that EGCG suppresses NET formation by downregulating CXCL2, thereby reducing pulmonary inflammation 104. EGCG has demonstrated significant therapeutic benefits in LPS-induced ALI. Given the mechanistic similarities in the pathogenesis, it is plausible that EGCG could also be a promising treatment for RILI. Nevertheless, the efficacy of EGCG in treating RILI needs to be further investigated through additional experimental and clinical research.

Studies have shown that hypoxia-inducible factor-1α (HIF-1α) upregulates phosphofructokinase/fructose-2,6-bisphosphatase 2 (PFKFB2), which increases glycolysis, accelerates dendritic cell (DC) maturation, and amplifies immune activation. These processes exacerbate inflammatory responses and contribute to ALI progression. In a mouse model of LPS-induced ALI, both DC-specific PFKFB2 knockout and DC-targeted delivery of HIF-1α inhibitor-loaded nanoparticles effectively suppressed DC maturation and alleviated ALI severity 105. Calcitonin gene-related peptide (CGRP) has been shown to inhibit the HIF-1α pathway and modulate macrophage polarization balance 106. Additionally, ophiopogonin D (OP-D) improves pulmonary microvascular endothelial barrier dysfunction by targeting the HIF-1α-VEGF pathway, thereby mitigating LPS-induced ALI 107. While the HIF-1α pathway demonstrates significant therapeutic potential, its efficacy in treating radiation pneumonia requires further experimental validation.

Radiotherapy induces DNA damage, including mitochondrial DNA damage, underscoring the therapeutic potential of targeting mitochondria. Esketamine activates mitophagy via the ULK1/FUNDC1 signaling pathway and exhibits beneficial effects in LPS-induced ALI animal models. It improves lung vascular permeability, reduces inflammatory responses, apoptosis, and oxidative stress 108. The novel mitophagy inducer TJ0113 selectively targets damaged mitochondria, induces mitophagy, and suppresses the NF-κB pathway, thereby alleviating LPS-induced inflammation. However, its therapeutic potential for RP remains to be confirmed through further studies 109.

The Rap1 pathway is involved in cell proliferation, differentiation, and survival, and plays a significant role in immune regulation, angiogenesis, and cancer development. A research team has confirmed the therapeutic effects of anlotinib in RILI, and the authors additionally conducted KEGG enrichment analysis to reveal potential pathways, including Rap1, that may contribute to the treatment effects. However, this pathway lacks validation through *in vitro* and animal experiments 110. Liu and Wang et al. validated through animal experiments that Potentilla anserina L. polysaccharide (PAP) significantly reduced LPS-induced ALI inflammation and inhibited M1 macrophage immune responses by activating the Rap1 signaling pathway 111. Prostaglandins are an important group of lipid mediators with barrier-protective potential towards the vascular endothelium. Prostacyclin (PC) exhibits potent protective effects in ischemia-reperfusion and ventilator-induced lung injury. Experimental evidence shows that post-treatment with PC activates the Epac/Rap1/afadin-dependent mechanism, promoting endothelial barrier repair and reducing p38 MAPK and NF-κB inflammatory pathways, thereby accelerating lung recovery 112. PC has been shown to effectively treat LPS-induced ALI in experimental models. Given its mechanisms, it may also hold therapeutic promise for RILI. However, its effectiveness in this setting needs to be rigorously evaluated.

Vasodilator-stimulated phosphoprotein (VASP) is a crucial controller of cytoskeletal dynamics and is essential for processes including cell migration, cytoskeletal restructuring, and the regulation of inflammatory responses. VASP demonstrates anti-inflammatory properties in liver tissues, but in lung tissues, its deletion significantly reduces symptoms in mice with LPS-induced ALI. VASP knockdown protects against LPS-induced ALI in mice by inhibiting M1 macrophage polarization, with its protective effects partially mediated by the cGMP-PKG signaling pathway 113. The efficacy of targeting the cGMP-PKG signaling pathway in treating RILI remains unclear.

Macrophage efferocytosis is integral to immune system function, playing a vital role in preserving tissue equilibrium, mitigating inflammation, and facilitating healing. Research indicates that sorafenib, a selective ADAM9 inhibitor, can enhance ALI symptoms and is crucial in modulating inflammatory responses 114. This inhibition leads to a decrease in macrophage and neutrophil counts in bronchoalveolar lavage, a reduction in pro-inflammatory cytokine levels, and an elevation of anti-inflammatory cytokines. Additionally, *in vitro* studies reveal that reducing ADAM9 expression boosts macrophage efferocytosis of apoptotic PMNs. The team also confirmed that ADAM9 in BMDMs binds to ITGAV in PMNs, and that inhibiting ITGAV enhances the macrophage efferocytosis mediated by ADAM9. Moreover, stifling the interaction between ADAM9 and ITGAV could potentially lead to the improvement of LPS-induced ALI by stimulating macrophage efferocytosis. Currently, there is a lack of validation regarding the efficacy of the ADAM9/ITGAV pathway in the treatment of RILI.

Currently, the burgeoning research into traditional Chinese medicine is fostering increased public recognition. Xuebijing Injection (XBJ), a preparation based on the Hematopoietic Blood Stasis Dispelling Soup formula, has exhibited potential in various therapeutic applications. The studies by Cui et al. have highlighted its capacity to significantly reduce plasma levels of endothelial cell damage-related biomarkers in LPS-induced sepsis and to mitigate LPS-induced lung injury by inhibiting the ACLY/MYB/RIG-I pathway 115. Nevertheless, the question of whether XBJ can also effectively reduce endothelial damage in the context of RILI has yet to be answered, with its therapeutic promise in this domain requiring further exploration and validation.

**Figure 3:**
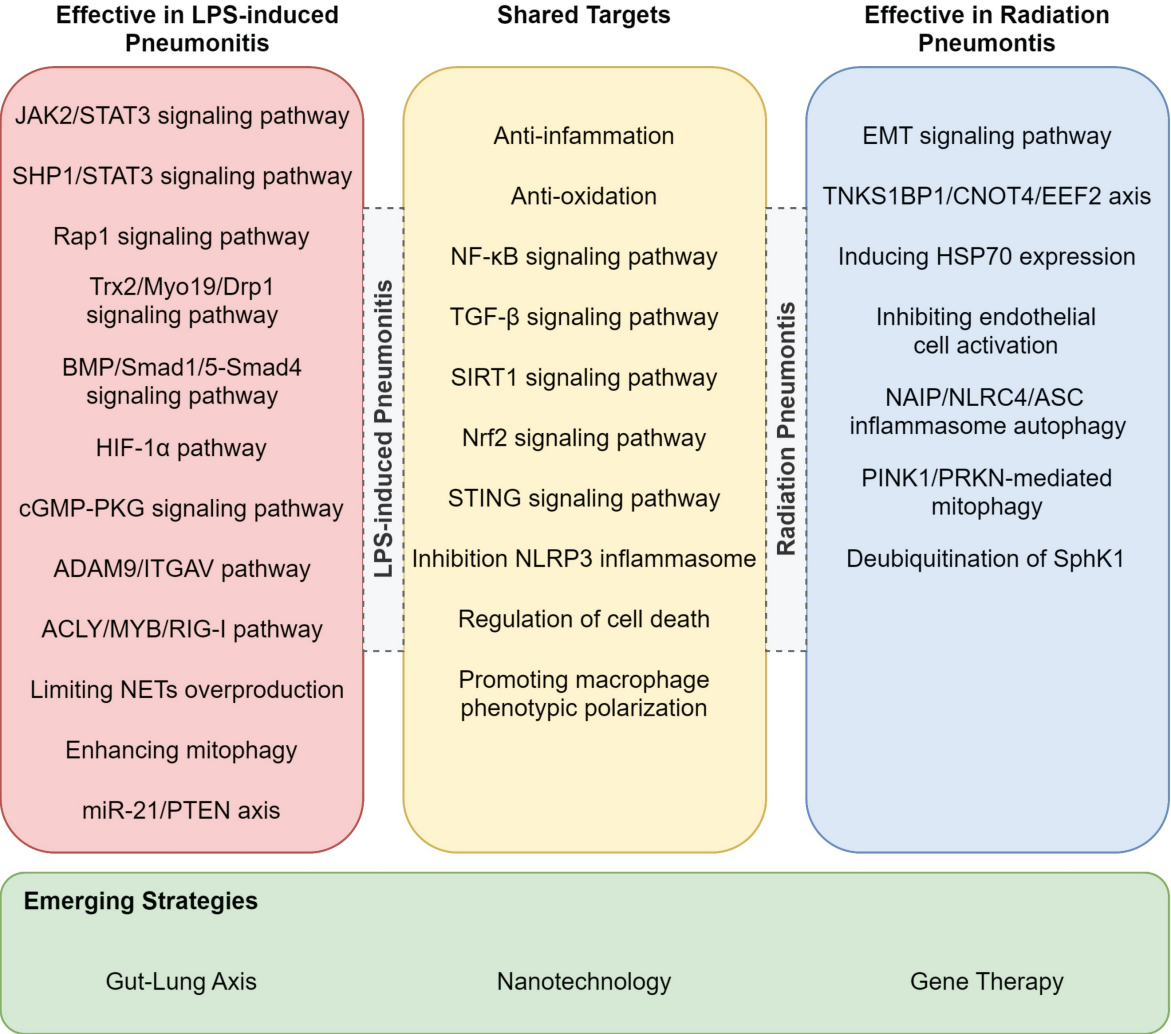
** Potential treatments for RP or LPS-induced pneumonitis.** This figure lists pathways validated for treating LPS-induced and radiation pneumonitis, highlighting shared targets and emerging strategies.

While RP and LPS-induced pneumonitis diverge in etiology and therapeutic focus, their shared inflammatory endpoints (e.g., alveolar injury, cytokine storm) reveal a hidden therapeutic synergy. For example, TGF-β-targeted antifibrotics for RP could mitigate late-stage fibrosis in LPS-ALI, whereas TLR4 antagonists for LPS-ALI might attenuate radiation-induced bystander effects 116. The treatments listed in Table 3, though currently used exclusively for either RP or LPS-ALI, may hold dual efficacy through overlapping pathways-a hypothesis awaiting experimental validation. In addition to the previously mentioned target pathways, we have also identified that drugs can alleviate LPS-induced lung injury in mice through mechanisms such as the STAT3 signaling pathway and miR-21/PTEN axis **(Figure 3)** 111, 117-121. Whether these pathways could serve as potential therapeutic targets for RP remains to be further investigated. Furthermore, other pathways such as NAIP/NLRC4/ASC inflammasome autophagy, PINK1/PRKN-mediated mitophagy, and the TNKS1BP1/CNOT4/EEF2 axis have already been validated as effective targets for treating RP **(Figure 3)** 122-127.

## Conclusion and perspectives

RP and LPS-induced pneumonitis exhibit similar manifestations, including alveolar damage and inflammatory infiltrates but they have different molecular mechanisms and clinical managements. RP primarily results from radiation-induced DNA damage, oxidative stress, and dysregulated TGF-β signaling, which collectively contribute to the development of pulmonary fibrosis. In contrast, LPS-induced pneumonitis is triggered by TLR4-dependent inflammatory signaling, characterized by a cytokine storm and a neutrophil- predominant immune response. Notably, both diseases share key pathways such as the release of certain inflammatory mediators and oxidative stress, suggesting that therapeutic strategies targeting these common nodes may be beneficial for both conditions, offering a potential for unified treatment approaches. In this comprehensive review, we have explored the therapeutic potential of RP by investigating a variety of approaches, including anti-inflammatory, antioxidant strategies, diverse modes of cell death, gut microbiota modulation, gene therapy, and nanotechnology. Our analysis has unveiled a subset of drugs that, while primarily employed in the treatment of LPS-induced pneumonitis, show considerable promise for RP by targeting shared mechanisms. These findings not only enrich our understanding of the therapeutic landscape for RP but also provides critical guidance for developing innovative drug repurposing strategies.

## Figures and Tables

**Table 1:** Clinical management for RP and LPS-induced pneumonitis.

Category	Radiation Pneumonitis	LPS-induced pneumonitis
**Diagnostic Criteria**	- Mandatory: History of thoracic radiotherapy - Imaging: Ground-glass opacities confined to radiation field (CT) - Clinical presentation	- Mandatory: Bacteriological evidence (e.g.,Bacterial culture/smear) - Imaging: Diffuse alveolar damage (HRCT)
**Conventional Treatment**	- High-dose Corticosteroids - Supportive oxygen therapy - Immunosuppressants: Azathioprine, mycophenolate - Antifibrotic agents: Pirfenidone/Nintedanib (for chronic progression)	- Combination or monotherapy of pathogen-sensitive antibiotics - Low-dose corticosteroids - Supportive oxygen therapy - Conditional use of antifibrotic agents
**Immunity Modulation**	- Immunosuppressed patients: Avoid prolonged steroids; prioritize antifibrotics	- Neutropenic patients: Augment with granulocyte transfusions
**Preventive Strategies**	- Reducing radiation-induced toxicity: IMRT and VMAT - Prophylaxis: Amifostine (limited evidence)	- LPS exposure control: Environmental decontamination - Prophylaxis: Probiotics
**Emerging Therapies**	- Stem cell therapy: Mesenchymal stromal cells (MSCs) for fibrosis reversal (limited evidence)	- Anti-LPS vaccines - Microbiome modulation: Fecal microbiota transplantation

**Table 2:** Similarities and distinctions in cellular and molecular mechanisms between RP and LPS-induced pneumonitis.

Category	Features	Radiation Pneumonitis	LPS-induced Pneumonitis	References
**Inflammatory Response**	Inflammasome	NLRP3 inflammasome		48, 128
	Key cytokines/chemokines	TNF-α, TGF-β, IL-1β, IL-6, IL-8, IL-10, PDGF	TNF-α, IL-1β, IL-6, IL-8, IL-18	129, 130
**Oxidative Stress**	Antioxidant defense	Nrf2 activation		52, 131
	Major ROS sources	Direct ionizing radiation-induced water radiolysis; mitochondrial electron transport chain disruption	TLR4/NF-κB-driven NOX activation	20, 50
**Triggers**	Mechanistic cascade	Radiation → DNA damage/DAMPs → Sterile inflammation	LPS →TLR4 → Anti-pathogen immunity	132, 133
**Signaling Pathways**	Dominant pathways	NF-κB signaling pathway MAPK signaling pathway		134, 135
		ATM-Chk2-p53 signaling pathway TGF-β/Smad signaling pathway	USP7/MAPK14 axis Rho A/ROCK1/2 signaling pathway	74, 136-138
**Death of cells**	Primary modes	Pyroptosis, poptosis, ferroptosis, NETosis		86, 88
		Senescence, autophagy	Necroptosis	80

**Table 3:** Potential mechanism or key pathway for RP or LPS-induced pneumonitis

Mechanism of action	Treatment	Targets of action		References
**TGF-**β **signaling pathway**	Ergothioneine	LPS-ALI*	Reported: TGF-β/smad/snail signaling pathway	139
		RILI**	Not reported	
	Pirfenidone	LPS-ALI*	Reported: TGF-β/Smad signaling pathway	28, 140
		RILI**	Reported: TGF-β1/Smad3 pathway	
	Nicaraven	LPS-ALI*	Not reported	141
		RILI**	Reported: TGF-β/pSmad2 pathway	
**Nrf2 signaling pathway**	Penehyclidine hydrochloride (PHC)	LPS-ALI*	Reported: mTOR / keap1 / Nrf2 signaling pathway	142
		RILI**	Not reported	
	Anisodamine	LPS-ALI*	Not reported	55
		RILI**	Reported: Nrf2/ARE signaling pathway	
	Pyrroloquinoline quinone (PQQ)	LPS-ALI*	Not reported	143
		RILI**	Reported: the MOTS-c/Nrf2 signaling pathway	
**NF‑κB signaling pathway**	Dihydroartemisinin	LPS-ALI*	Reported: NF‑κB signaling pathway	144, 145
		RILI**	Reported: cGAS/STING/ NF‑κB signaling pathway	
	Ethyl caffeate	LPS-ALI*	Reported: TNF-α/NF-κB/MMP9 axis	146
		RILI**	Not reported	
	3,3'-Diindolylmethane	LPS-ALI*	Not reported	147
		RILI**	Reported: TGF-β/Smad and NF-κB dual pathways	
**NLRP3 signaling pathway**	Glycyrrhizin	LPS-ALI* & RILI**	Reported: Inhibiting the NLRP3 inflammasome	148, 149
	Amentoflavone (AF)	LPS-ALI*	Reported: NLRP3/ASC/Caspase-1 axis	89
		RILI**	Not reported	
	Raspberry ketone	LPS-ALI*	Not reported	150
		RILI**	Reported: STAT2-P2X7r/NLRP3 pathway	
**AMPK signaling pathway**	Hydrogen-rich solution	LPS-ALI*	Reported: ROS/AMPK/mTOR pathway	151, 152
		RILI**	Reported: AMPK/mTOR/ULK1 signaling pathway	
	Nerolidol	LPS-ALI*	Reported: AMPK/Nrf-2/HO-1 pathway	153
		RILI**	Not reported	
**SIRT1 signaling pathway**	L‑carnitine	LPS-ALI*	Not reported	154
		RILI**	Reported: AMPK/SIRT1/TGF‑1ß pathway	
	Dapagliflozin (DPG)	LPS-ALI*	Reported: SIRT-1/PGC-1α pathway	90
		RILI**	Not reported	
**HIF-1α signaling pathway**	Calcitonin gene-related peptide (CGRP)	LPS-ALI*	Reported: Inhibiting the HIF-1α pathway	106
		RILI**	Not reported	
	Ophiopogonin D (OP-D)	LPS-ALI*	Reported: the HIF-1α-VEGF pathway	107
		RILI**	Not reported	
**Rap1 pathway**	Potentilla anserina L. polysaccharide (PAP)	LPS-ALI*	Reported: the Rap1 signaling pathway	111
		RILI**	Not reported	
	Prostacyclin (PC)	LPS-ALI*	Reported: the Epac/Rap1/afadin-dependent mechanism & reducing p38 MAPK and NF-κB inflammatory pathways	112
		RILI**	Not reported	
**cGMP-PKG signaling pathway**	VASP knockdown	LPS-ALI*	Reported: Inhibiting M1 macrophage polarization mediated by the cGMP-PKG signaling pathway	113
		RILI**	Not reported	
**ACLY/MYB/RIG-I pathway**	Xuebijing Injection (XBJ)	LPS-ALI*	Reported: Inhibiting the ACLY/MYB/RIG-I pathway	115
		RILI**	Not reported	
**Death of cells**	Uridine	LPS-ALI*	Reported: Inhibiting ferroptosis of macrophage	155
		RILI**	Not reported	
	Astragaloside IV	LPS-ALI*	Not reported	156
		RILI**	Reported: Suppressing ferroptosis	
**Anti-oxidation**	Curcumin	LPS-ALI*	Reported: Enhancing anti-oxidant levels	157, 158
		RILI**	Reported: Free radical scavenging and anti-oxidation	
	Alpinumisoflavone	LPS-ALI*	Reported: Anti-oxidation and anti-inflammation	159
		RILI**	Not reported	
	Suplatast tosilate	LPS-ALI*	Not reported	160
		RILI**	Reported: Suppression of oxidative stress	
**Anti-inflammatory**	L-carnitine	LPS-ALI*	Reported: Mitochondria modulation and inflammation control	154, 161
		RILI**	Reported: the AMPK/SIRT1/TGF‑1ß pathway	
	Diethylcarbamazine	LPS-ALI*	Reported: Apoptosis induction for inhibiting inflammatory cell accumulation	162, 163
		RILI**	Reported: Suppressing COX-2 and NF-kB pathway	
**Regulation of gut microbiota**	Cordyceps militaris solid medium extract (CMME)	LPS-ALI*	Reported: Regulating intestinal flora and correcting metabolic disorders	94
		RILI**	Not reported	
	Faecal microbiota transplantation (FMT)	LPS-ALI*	Not reported	96
		RILI**	Reported: Improving GI tract function and epithelial integrity	
**Limiting NETs overproduction**	Epigallocatechin-3-gallate (EGCG)	LPS-ALI*	Reported: Downregulating CXCL2	104
		RILI**	Not reported	
**Enhancing mitophagy**	Esketamine	LPS-ALI*	Reported: the ULK1/FUNDC1 signaling pathway	108
		RILI**	Not reported	
	TJ0113	LPS-ALI*	Reported: inducing mitophagy and suppressing the NF-κB pathway	109
		RILI**	Not reported	
**Macrophage efferocytosis**	sorafenib	LPS-ALI*	Reported: the ADAM9/ITGAV pathway	114
		RILI**	Not reported	

*: Including LPS-induced ALI, LPS-induced pneumonitis, LPS-induced ARDS. **: Including RILI, RP, RIPF

## Abbreviations

OH: hydroxyl radicals
ACSL4: acyl-CoA synthetase long-chain family member 4
AF: Amentoflavone
ALI: acute lung injury
ALOXs: arachidonate lipoxygenases
ARDS: acute respiratory distress syndrome
ASMB: Au@mSiO2@Mn(CO)5Br
CMME: Cordyceps militaris solid medium extract
CTCAE: Common Terminology Criteria for Adverse Events
Cur@HMSN-BSA: Curcumin-loaded ROS-responsive hollow mesoporous silica nanoparticles
DAMPs: Damage-associated Molecular Patterns
DDR: DNA damage response
DPG: Dapagliflozin
DSBs: double-strand breaks
EMT: Epithelial-mesenchymal transition
EndMT: endothelial-mesenchymal transition
FMT: faecal microbiota transplantation
GPX4: glutathione peroxidase 4
GSH: glutathione
H₂O₂: hydrogen peroxide
IKKε: IκB Kinase ε
IRF3: Interferon Regulatory Factor 3
LBP: LPS binding protein
LOOHs: lipid hydroperoxides
LPS: lipopolysaccharide
MAPK: mitogen-activated protein kinase
MD2: Myeloid differentiation protein-2
MnSOD: Manganese superoxide dismutase
MODS: multiple organ dysfunction syndrome
MSCs: mesenchymal stem cells
mtDNA: mitochondrial DNA
mtROS: mitochondrial ROS
MyD88: myeloid differentiation primary response 88
NETs: neutrophil extracellular traps
NF-κB: nuclear factor κB
NLRP3: NLR Family Pyrin Domain-Containing 3
NOX: NADPH oxidase
Nrf2: Nuclear factor erythroid 2-related factor 2
NSAIDs: non-steroidal anti-inflammatory drugs
OB: Obacunone
PAR: poly ADP-ribose
PMNs: polymorphonuclear neutrophils
pMnSOD: plasmid-mediated MnSOD
PRRs: pattern recognition receptors
PUFAs: polyunsaturated fatty acids
RILI: radiation induced lung injury
RIPF: radiation induced pulmonary fibrosis
ROS: reactive oxygen species
RP: radiation pneumonitis
RTOG: Radiation Therapy Oncology Group
SASP: senescence-associated secretory phenotype
SOD: superoxide dismutase
SSBs: single-strand breaks
TBK1: TANK-Binding Kinase 1
TIR: toll/interleukin-1 receptor
TLR: Toll-like receptor
TRAF: TNF Receptor-Associated Factor
TRAM: Tumor Necrosis Factor Receptor-Associated Machinery
TRIF: TIR-domain-containing adapter inducing interferon-β
